# Behaviour of Prestressed CFRP Anchorages during and after Freeze-Thaw Cycle Exposure

**DOI:** 10.3390/polym10060565

**Published:** 2018-05-23

**Authors:** Yunus Emre Harmanci, Julien Michels, Eleni Chatzi

**Affiliations:** 1 Institute of Structural Engineering, Department of Civil, Environmental & Geomatic Engineering, ETH Zürich, 8049 Zürich, Switzerland; chatzi@ibk.baug.ethz.ch; 2 Structural Engineering Research Laboratory, Swiss Federal Laboratories for Materials Science and Technology, Empa, 8600 Dübendorf, Switzerland; Julien.Michels@empa.ch; 3 re-fer AG, 6440 Brunnen, Switzerland

**Keywords:** structural strengthening, CFRP, prestressing, epoxy, bond behavior, fiber optic sensors, finite element modeling, durability, swelling, freeze-thaw-cycles

## Abstract

The long-term performance of externally-bonded reinforcements (EBR) on reinforced concrete (RC) structures highly depends on the behavior of constituent materials and their interfaces to various environmental loads, such as temperature and humidity exposure. Although significant efforts have been devoted to understanding the effect of such conditions on the anchorage resistance of unstressed EBR, with or without sustained loading, the effect of a released prestressing has not been thoroughly investigated. For this purpose, a series of experiments has been carried out herein, with concrete blocks strengthened with carbon fiber-reinforced polymer (CFRP) strips, both unstressed, as well as prestressed using the gradient anchorage. The gradient anchorage is a non-mechanical technique to anchor prestressed CFRP by exploiting the accelerated curing property of epoxy under higher temperatures and segment-wise prestress-force releasing. Subsequently, strengthened blocks are transferred into a chamber for exposure in dry freeze-thaw cycles (FTC). Following FTC exposure, the blocks are tested in a conventional lap-shear test setup to determine their residual anchorage resistance and then compared with reference specimens. Blocks were monitored during FTC by conventional and Fabry–Pérot-based fiber optic strain (FOS) sensors and a 3D-digital image correlation (3D-DIC) system during gradient application and lap-shear testing. Results indicate a reduction of residual anchorage resistance, stiffness and deformation capacity of the system after FTC and a change in the failure mode from concrete substrate to epoxy-concrete interface failure. It was further observed that all of these properties experienced a more significant reduction for prestressed specimens. These findings are presented with a complementary finite element model to shed more light onto the durability of such systems.

## 1. Introduction

**Figure 1:**
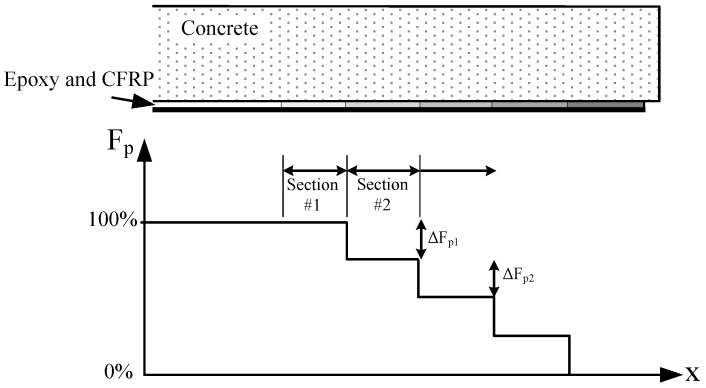
The concept of gradient anchorage, where each section comprises a heating and partial prestress-force-release step.

The use of fiber-reinforced polymers (FRP), particularly those comprising carbon fibers (CFRP), has anchored its position within researchers and practitioners as an effective retrofitting technique for reinforced concrete (RC) flexural members. To this day, the most common strengthening approach is the application of either fabrics or pultruded strips on the tensile face of the member, often referred to as externally-bonded reinforcement (EBR). CFRP has excellent mechanical properties (E>150GPa, $f_{u}>2500\mathrm{MPa}$); however. most of the time, the full potential of this material cannot be exploited with the EBR technique due to premature debonding of CFRP [1]. Prestressing is a potential method to postpone, or in some cases even eliminate, the abrupt debonding failure mode and allow the flexural member to fail either from CFRP tensile failure or conventional concrete crushing. In prestressed systems, the end-anchorage is arguably the most critical section since concrete cannot carry the high shear stresses at the strip-end on its own [2]. A number of metallic and non-metallic mechanical anchorage techniques have been developed and are in use today within the industry [3]. As an alternative to mechanical approaches, gradient anchorage is a non-mechanical technique developed at Empa (www.empa.ch) to allow for potentially faster installation times without the need for any metallic or non-metallic components [4]. This method exploits the accelerated curing property of epoxy via external heating and only relies on the constituents of the strengthening system, i.e., concrete, epoxy resin and the CFRP strip. The main principle of this technique is to avoid premature delamination by multiple sections of segment-wise heating and prestress-force-releasing at the strip ends, limiting the interfacial shear stress that occurs during releasing [5]. This concept is qualitatively visualized in Figure 1, where it can be seen that after accelerated curing of the first section, the prestressing force $F_{p}$ is partially released by $\Delta F_{p1}$. This procedure is sequentially repeated until zero prestressing force remains at the strip end. The released prestressing force and section length are selected to ensure concrete can carry the associated shear stresses, which is the actual relevant design parameter. Strengthened flexural members anchored with the gradient method were demonstrated to perform comparably to their mechanical variants [6,7], even some cases reaching CFRP tensile failure without debonding [1]. Further information on the anchorage itself can be found in [8]. The interfacial shear stress, namely the prestressing force, used in this study is selected based on the aforementioned studies, as well as previous work at Empa.

The studies given above solely focus on the short-term behavior of the anchorage, i.e., kept within ambient lab conditions and tested shortly after application. Infrastructures are exposed to a variety of environmental effects throughout their lifetime, such as changes in temperature and humidity, and it is thus crucial for retrofitting methods to ensure a safe performance during and after such exposures. As with conventional unstressed EBR techniques, prestressed EBR (PEBR) with gradient anchorage solely relies on its constituents, that is concrete, epoxy and CFRP. The long-term behavior of these constituents and their interactions need a thorough investigation to guarantee a safe performance over the design life. At Empa, the long-term behavior of a CFRP-strengthened concrete structure is currently a core research topic [9,10]. It was, among other findings, shown that the installation of the warm mastic asphalt layer might cause a temporary stiffness reduction in the epoxy adhesive and hence lead to a prestress loss in the case of a gradient anchorage. In such a case, a protection system is required. Initially unstressed CFRP strips are not affected in a negative way. In the long-term, a combination of sustained load and exposure to seasonal temperature changes has up to today not shown negative consequences for either unstressed or prestressed strengthening systems. Even the temperature rise in the epoxy layer due to heating up of the mastic asphalt layer under direct sun irradiation has not provoked any significant changes in the strain state or relevant damages. Furthermore, fatigue loading has been included in the investigations, as well [11]. In parallel, the durability of both mechanical and gradient anchorages for prestressed CFRP strips after immersion in water and chlorides has been studied at the University of Minho [12]. The results have shown that immersion in a water with or without chloride content, with a simultaneously-sustained load or not, leads to slightly lower residual load carrying capacities and structural ductility compared to the reference specimens. It was concluded that the immersion period should be extended in order to assess any further possible decrease in performance. An overall summary of these findings can be found in Sena-Cruz et al. (2017) [13].

In this study, an experimental campaign was launched to investigate the effect of common environmental deterioration phenomena, such as carbonation and freeze-thaw cycles (FTC). Results have shown that carbonation bears a positive effect through the increased strength of concrete [14], yet FTC causes a dramatic decrease of the residual anchorage resistance and deformation capacity (considering the maximum slip at failure) and also promotes a shift in the failure mode, from the conventional concrete-substrate to an epoxy-concrete interface failure [15,16].

Although the effect of FTC on the residual anchorage resistance of gradient anchorage was quantified within the previous study, the underlying mechanisms causing the deterioration and shift in failure mode remain unknown. With the current knowledge, it is assumed that the deterioration initiates and propagates due to chemical and mechanical processes [15,17]. Another important question that remains unanswered is whether FTC effects PEBR differently than EBR. This has been only partially tackled through studies considering sustained loading on the system during environmental exposure [18]. These results cannot be directly inherited to decisively conclude about the gradient anchorage since its loading mechanisms on the bond are shown to be different [5]. With this study, these two important open questions are tackled experimentally.

## 2. Materials and Methods

### 2.1. Experimental Investigation

**Figure 2:**
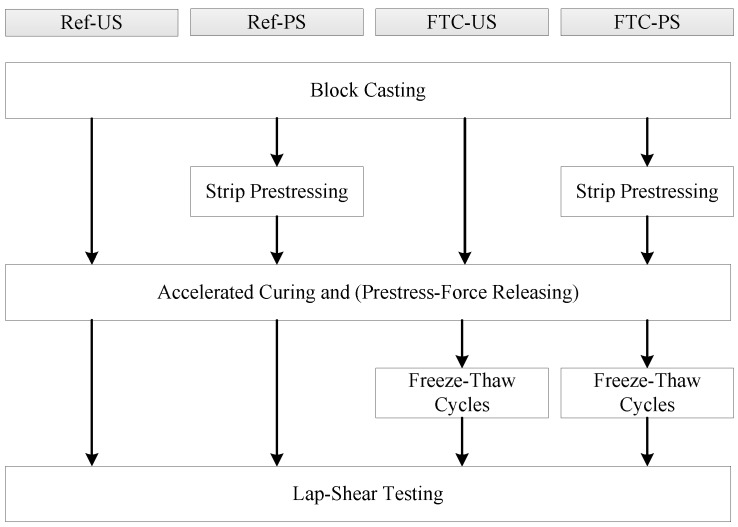
Chart summarizing key steps for each testing scenario of the experimental campaign.

In order to investigate the effect of prestressing and FTC on the residual anchorage resistance, four scenarios were considered for the experimental campaign. Two reference scenarios, one unstressed (Ref-US) and one prestressed (Ref-PS), provide the necessary benchmark behavior of the anchorage in the short-term time scale. Subsequently, identically-constructed blocks were subjected to freeze-thaw cycles, henceforth referred to analogously as FTC-US and FTC-PS. Three specimens were prepared for each scenario, and their key steps for each scenario are summarized and provided in Figure 2.

#### 2.1.1. Specimen Preparation

**Figure 3:**
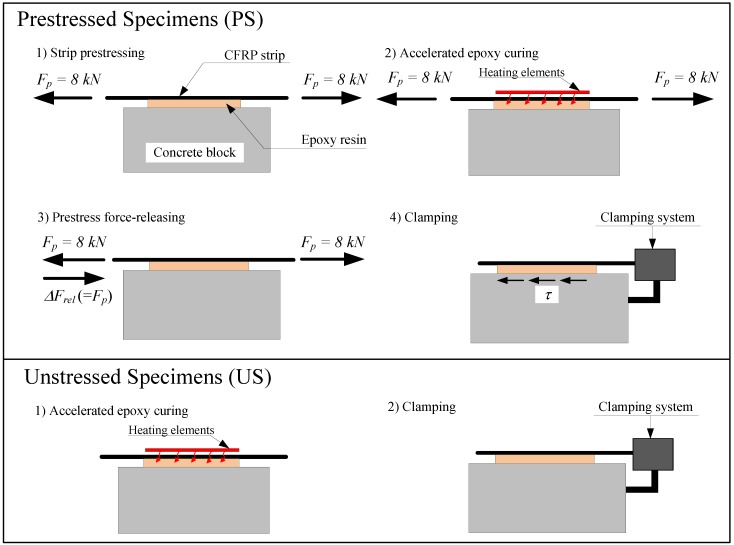
Key steps of specimen preparation for prestressed (**top**) and unstressed (**bottom**) blocks.

A Portland-limestone cement (CEM II/A-LL 42.5 N) was used with well-rounded alluvial gravel ($d_{max}$ = 16 mm), a water-cement ratio of 0.48 and without air entraining admixture. A commercially available thixotropic two-component epoxy (S&P 220 [19]) was used to bond the CFRP strips to the concrete blocks. As visualized in Figure 1, the gradient anchorage is realized in multiple segments to safely release the prestressing force towards the strip-ends. In order to minimize uncertainties pertaining to the interactions between neighboring segments, a single segment anchorage was constructed for the experimental investigation. This is achieved via prestressing a 50 × 1.2 mm pultruded CFRP strip (S&P Clever Reinforcement [20]) with 8 kN of force, which corresponds to roughly 1.1 MPa of bond shear stress over an area of 150 × 50 ${\mathrm{mm}}^{2}$ if a constant stress distribution is assumed. The experimentally-achieved prestressing forces ranged between 8.00 and 8.29 kN, which translates to average bond shear stress values between 1.07 and 1.11 MPa. The CFRP surface is then heated by a specially-designed heating device with a pre-defined heating regime and left for cooling. After reaching an epoxy temperature of roughly $25{\;}^{\circ }$C following cooling, the prestressing force is released from one strip end, transferring shear forces onto the bonded area. The opposite end of the anchorage holding the prestressing force after releasing is supported by a custom clamping system, which allows the block to be transported to the climate chamber while maintaining the prestressing force fairly constant. Unstressed specimens undergo the same heating protocol for proper comparison, yet they do not comprise a prestressing and force releasing step. Nevertheless, the unstressed specimens are still equipped with the clamping system due to the ease of application prior to accelerated aging exposure, as well as to embody an identical specimen configuration as the prestressed cases. A graphical summary of this procedure is given in Figure 3. Readers that are interested in the specific details of specimen preparation are suggested to refer to [15].

**Figure 4:**
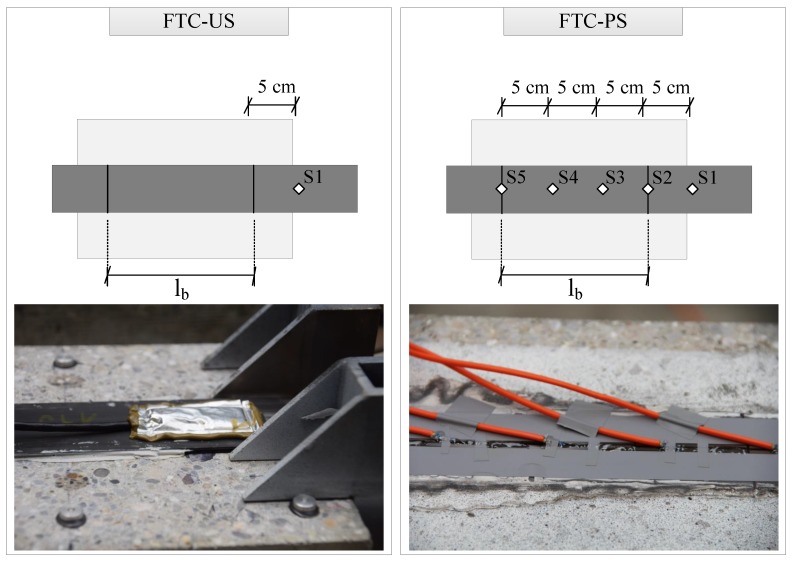
**Left**: Conventional strain sensor placed at the free-length for unstressed blocks; **right**: fiber optic strain sensors placed at the free-length and along the bonded region for prestressed blocks.

Following the preparation of both unstressed and prestressed blocks, a set of strain sensors was installed on the blocks that are undergoing freeze-thaw cycles, as presented in Figure 4. The stress state of the system during FTC is crucial for a complete understanding of the effect of this accelerated aging exposure. It is, however, not possible to deploy the DIC system in the freeze-thaw chamber, and thus, such point-wise measurements are needed. Unstressed blocks are equipped with a conventional strain gauge (HBM GmbH), placed at the free-length, at a 5-cm distance from the bonded region. Prestressed blocks are equipped with Fabry–Pérot white light interferometry-based fiber optic strain sensors (FISO), with five sensors per block placed evenly every 5 cm, starting from the released end of the bond until the free length at a 5-cm distance from the bonded end. Strain gauges were protected as per the producer’s recommendation with a covering foil (ABM75, HBM) to provide a barrier against moisture attack. Fiber optic sensors (FOS) were attached to the CFRP strip with a two-component adhesive according to the producer’s recommendations (M-Bond AE10, Vishay) without any additional moisture protection since these sensors are immune to such effects. Additionally, no dummy sensor is needed for the Fabry–Pérot-type sensors since they are temperature independent and do not require compensation. The obtained strain readings are consequently used to assess the strain evolution of the anchorage system during FTC, which could shed light onto any potential damage/relaxation effect.

Furthermore, 5 unstrengthened concrete blocks from the same batch were deployed with deformeter bolts to measure the shrinkage/swelling behavior of concrete itself in ambient and varying RH conditions. For this purpose, blocks were taken out of storage (90% RH) and stored for two weeks (approximate time the strengthened blocks have spent before initiating FTC) at ambient conditions ($20{\;}^{\circ }$C, 35% RH). Subsequently each block was transferred to a separate climate chamber with constant temperature ($20{\;}^{\circ }$C) and varying RH levels (36%, 57%, 70%, 90%, 100% RH) for 8 weeks, which corresponds to the duration of FTC. Lastly, they are once again stored in ambient lab conditions for two weeks, which corresponds to the approximate time the blocks spent between FTC and lap-shear testing. Strains along the length of each block were measured regularly to obtain shrinkage and swelling information during each of these periods.

#### 2.1.2. Accelerated Aging Procedure

The freeze-thaw cycles employed herein were designed specifically for this study since it was deemed that conventional testing procedures used for concrete such as the ASTM C666-03 [21] are too extreme and unrealistic for the anchorage. A modified version of Swiss SIA 262/1:2013-08 [22] is adopted herein, where the cycle times are modified and thawing by wetting eliminated, once again due to the unrealistic severity of these conditions on EBR.

**Figure 5:**
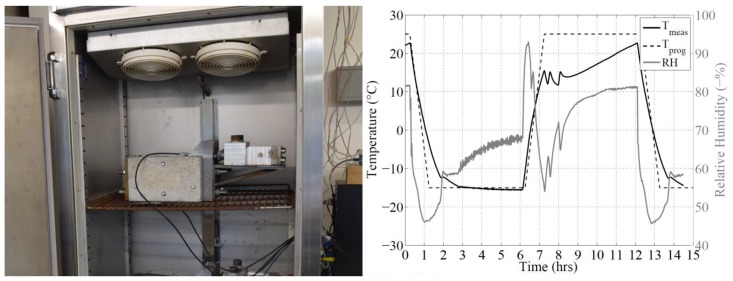
**Left**: FTC chamber employed within this study; **right**: programmed temperature, measured temperature and measured RH for one cycle, adopted from [16].

The concrete mix is designed to comply with the exposure class XF3 according to EN206-1 [23], a highly-saturated environment with no de-icing salts. Considering the fact that the target application of this anchoring technique is directed towards flexural strengthening of bridge girders, one can agree that the exposure class XF3 is adequate for this purpose. The strengthened blocks were placed in a chamber to undergo dry-FTC, in which dry implies that thawing was not achieved by flooding the chamber, but only via air temperature. The setup within the chamber, as well as observed temperature and humidity levels are given in Figure 5. One hundred twenty cycles were applied, with the air temperature ranging between −15 and +25 ${}^{\circ }$C. The duration of each cycle was 12 h, with 5 h of constant temperatures at both extremes and a 1-h transition time between each. The chamber used is temperature controlled, but humidity control was not possible. Condensation was observed starting at the thawing ramp of each cycle due to the relative humidity exceeding 90%. As a consequence, the top faces of the blocks were constantly in a wet state.

The aforementioned shrinkage/swelling tests were conducted with the intent to possibly exclude potential effects of time-dependent volumetric changes of concrete from the system (assuming CFRP and epoxy are not affected by such effects). Due to technical difficulties, the CFRP strains are not measured after the end of the cycles, so swelling data might provide some insights for the strains existing in the system just before lap-shear testing.

#### 2.1.3. Lap-Shear Testing

**Figure 6:**
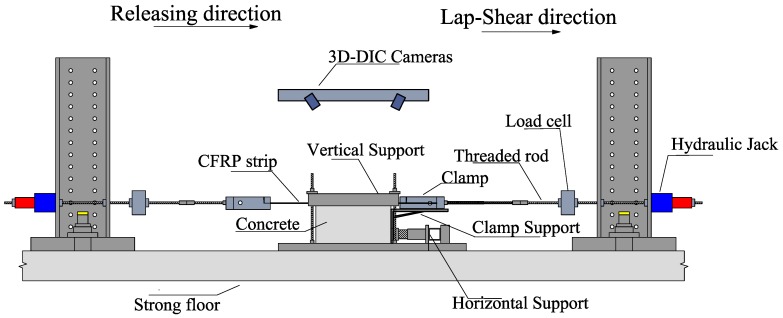
Schematic description of the lap-shear test setup and its key components. Adopted and modified from Michels et al. (2014) [24].

Conventional single-sided lap-shear tests are conducted at the end of each case to compare their bond behavior. The specimens were tested between the ages of 9–32 months after casting, where concrete is assumed to be mostly cured and no significant developments in material properties are expected to occur. The concrete block is supported at various locations to restrain the movement of the block while the CFRP strip is pulled. The aforementioned clamping system at the end of the strip is concentrically connected to a load cell and a hydraulic cylinder via a threaded rod. The hydraulic cylinder is connected to a pump, which is operated manually to increase the loading. The force is measured by a 150-kN load cell (burster GmbH, Gernsbach, Germany) and recorded with a 1-Hz sampling rate by a data acquisition system (HBM GmbH). A complete schematic description of the test setup is provided in Figure 6.

**Figure 7:**
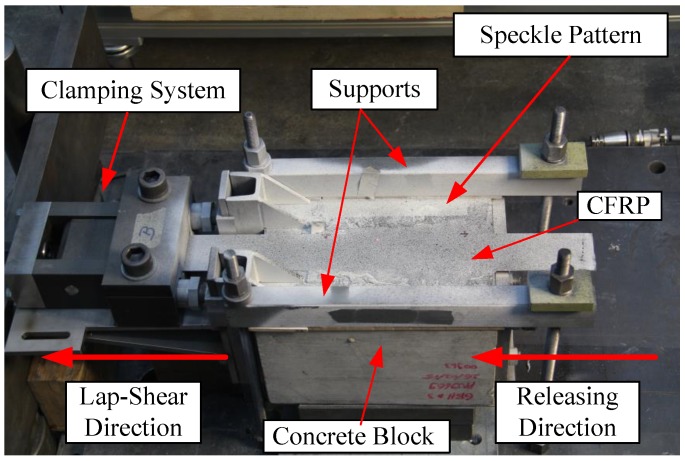
Lap-shear testing setup including the speckle pattern applied on the top surface of strengthened blocks.

Full-field displacements of the strengthened surface were measured with a 3D-DIC system (ARAMIS, gom GmbH) [25]. The measurement volume was selected as 250 × 250 × 250 ${\mathrm{mm}}^{3}$, and the images were captured with a 50-mm fixed focal length objective. Two 4-Megapixel sensors covered the region of interest, with a calibration deviation of 0.016 pixels. Every specimen was comprised of a black random speckle pattern over a white background, also visible in Figure 7 along with an overall view of the test setup.

**Figure 8:**
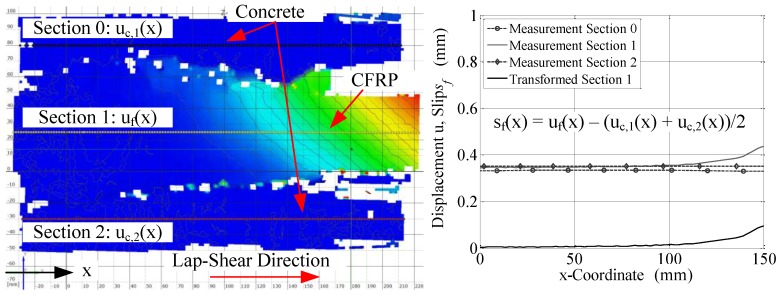
**Left**: Output of X-displacements (lap-shear loading direction) of a sample obtained from the test setup presented in Figure 6 and Figure 7, and the defined sections along the concrete and CFRP strip; **right**: displacements and obtained slip profile along the bond length, along with the formulation for slip derivation.

The images were then processed using the ARAMIS software, and raw displacements were extracted from sections of interest. The slip, obtained by subtracting average concrete displacements from CFRP displacements, was used for comparison purposes. The definition of slip is demonstrated in Figure 8, and a sample derivation was made using a random stage obtained during one of the lap-shear tests. It must be noted that the asymmetric displacement profile observed in Figure 8 (left) should not be interpreted as severe eccentricities introduced during lap-shear testing. As previously mentioned, the loading was applied as concentrically as possible; however, minor deviations occurred as expected due to movement at the supports and other geometric imperfections. Displacements provided in Figure 8 (right) prove this point, since the displacements along each section only deviate a fraction of the presented quantities.

### 2.2. Numerical Modeling

#### Modeling Approach

A finite element (FE) model was constructed in Abaqus to support experimental findings and infer more information about the behavior of the anchorage during and after FTC, at locations and instances where no experimental measurements were available.

**Figure 9:**
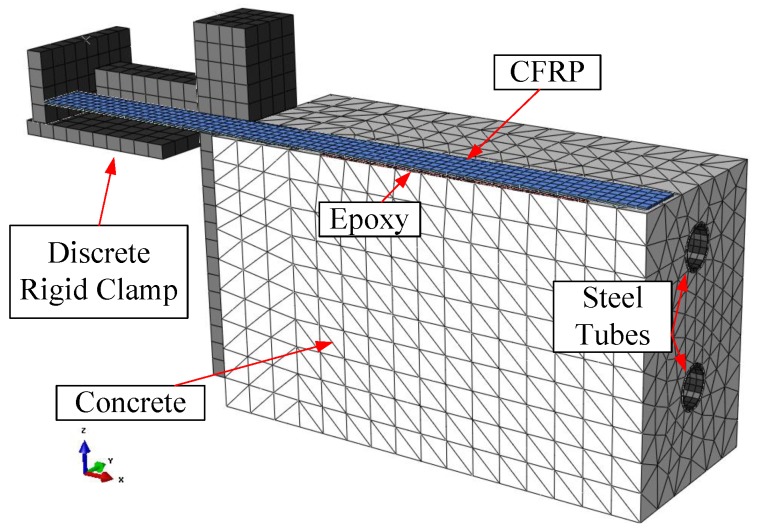
Finite element model constructed in Abaqus to simulate the time-dependent swelling and shrinkage behavior.

Due to the symmetric nature of the problem, only half of the system was modeled. Aside from the obvious constituents, all secondary components of the strengthened blocks were also modeled to capture the behavior as accurately as possible. These secondary components were namely the clamping system, modeled as a simplified set of discrete rigid blocks, and the steel tubes that were embedded in the concrete to attach the clamping system to the block. A screenshot of the constructed model is provided in Figure 9. Contact between the clamping system and the concrete block was realized by a hard normal contact and a frictional tangential contact formulation, whereas the steel tubes were simply tied. The CFRP strip was also tied to the epoxy layer, while the epoxy layer was attached to concrete with a cohesive contact law. The model consisted of ten-node tetrahedral elements (C3D10) and 8-node reduced integration brick elements with hourglass control. All materials were modeled as linear elastic since damage was not the focus of the simulations. Material properties for CFRP and the epoxy resin were adopted from the producer’s specifications [19,20]. The Young’s modulus of concrete was calculated to be approximately equal to 35 GPa according to the fib bulletin [26].

The volumetric changes experienced by concrete were introduced by a relatively simple method, i.e., by setting the coefficient of thermal expansion (CTE) to zero for every material except concrete, since only concrete was assumed to undergo swelling and shrinkage, and subsequently applying a temperature-time history to recreate the deformations induced by swelling under 100% RH exposure. Since no material degradation was modeled, even with the aforementioned contact definitions, the implicit (standard) solver of Abaqus with non-linear geometry (Nlgeom) enabled was sufficient to successfully calculate the structural response.

The loads were applied in two distinct steps, a step where the prestressing force was released from one end (only valid for PS), and a second step where the previously explained temperature-time-history was applied. The concrete block was restrained from moving during prestress-force-releasing by appropriate boundary conditions (BC) at the block base, as well as the top; however these BC’s were removed for the swelling/shrinkage simulations to allow for unconstrained volume change. The response of the system was obtained every 86,400 s, corresponding to daily measurements.

## 3. Results

### 3.1. Experimental Results

**Figure 10:**
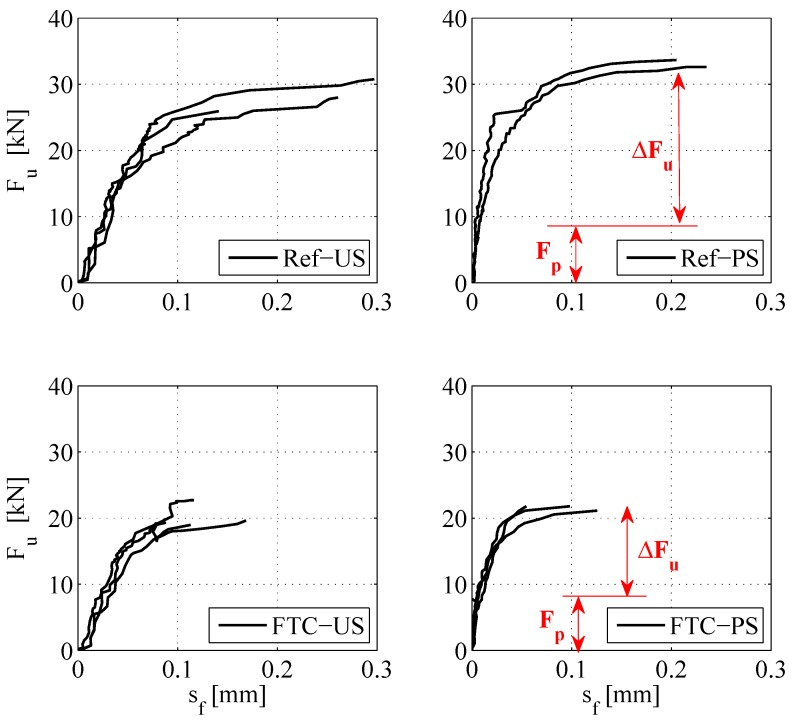
Force-slip relationship of all experiments.

The initial comparison to observe the effects of FTC was conducted based on the lap-shear behavior of strengthened blocks. Load-slip curves of each experiment are shown in Figure 10. It can be observed that both unstressed and prestressed reference specimens failed at an ultimate load ($F_{u}$) around 30 kN and a maximum slip ($s_{f,B}$) of roughly 0.2 mm. On the other hand, specimens subjected to freeze-thaw cycles failed at an ultimate load around 20 kN and a maximum slip of roughly 0.1 mm. Average values of each case are provided in Table 1. Firstly, it becomes evident that the maximum attained slip at failure decreased almost by half after FTC, 43% and 57%, respectively for the unstressed and prestressed cases, indicating that the reduction in deformation capacity was more pronounced in prestressed specimens.

The stiffness of the bond within the elastic range (defined by $s_{f,B,el}=0.02$ mm in [5]) can be determined by calculating the slope from the force-slip relationships in Figure 10, presented in Table 1. There was a significant increase in stiffness due to prestressing ($\frac{F_{el}}{s_{f,B,el}}$), which was expected. Prestressed specimens experience higher deteriorative effects due to FTC, since the reduction of stiffness in the elastic range decreases 9% and 32% for the unstressed and prestressed cases, respectively.

**Figure 11:**
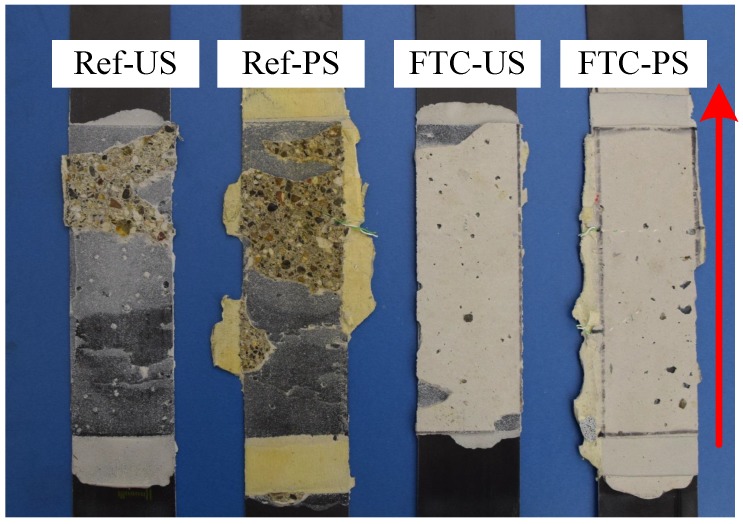
One sample from each case demonstrating the shift in the failure mode from a concrete-substrate to an epoxy-concrete interface failure.

The residual load carrying capacity, denoted by $\Delta F_{u}$, was used to compare the effect of prestressing, as well as FTC on the anchorage. The residual load carrying capacity was defined as the additional loading that was required, excluding the prestressing force, to bring the anchorage to failure. This implied that the prestressed case considered 8 kN (assuming no relaxation occurs) lower values for the residual resistance than the forces observed in Figure 10. The effect of prestressing can be better understood by comparing the residual anchorage resistance of unstressed and prestressed reference cases. As reported in Table 1, the residual anchorage resistances ($\Delta F_{u}$) of REF-US and REF-PS were 28.23 and 25.14 kN, respectively. The prestressed anchorage can eventually reach a higher ultimate load ($F_{u}$) of 33.14 kN; however, the residual load carrying capacity is lower due to the interaction of bond shear stresses of prestress-force-releasing and lap-shear testing. These findings are in line with experimental observations of Czaderski [5], who described this interaction as “the internal stresses from prestress force release consume a part of the fracture energy resistance of the surface concrete”. Moreover, an evident reduction exists in the residual load carrying capacity of the anchorage, following FTC. A reduction of 27% was obtained for the unstressed blocks, whereas a considerably higher reduction of 46% was observed for the prestressed blocks. As explained previously and reported in [15], the prestressed case experienced a shift in failure mode following FTC, from concrete substrate to an epoxy-interface failure. The identical phenomenon was also observed for the unstressed case, as shown in Figure 11. The reason for this shift was attributed to the effect of moisture on the chemical component of the bond between concrete and epoxy, as well as the effect of cyclic loading imposed by FTC on the mechanical component of the bond [27,28,29].

**Figure 12:**
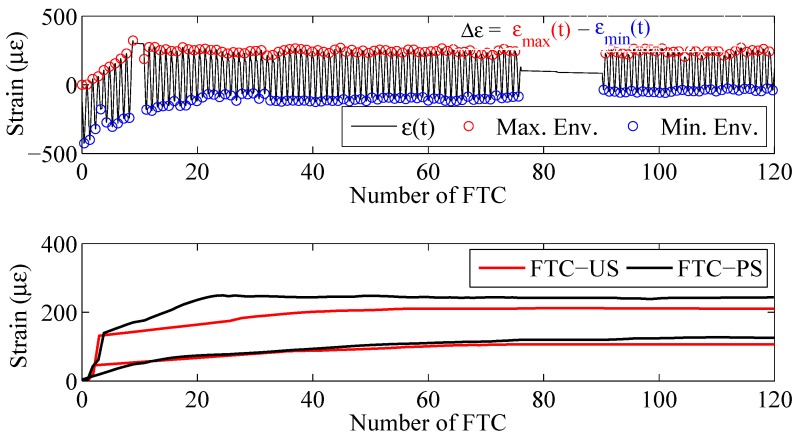
**Top**: Strain time-history of an FOS sensor placed at the free-length of a prestressed block, along with its maximum and minimum envelope during FTC; **bottom**: maximum strain envelope measured at the free-length during FTC for unstressed and prestressed blocks.

In addition to the lap-shear test results, measurements from strain sensors at the free-length (denoted as S1 in Figure 4) during FTC were processed and are discussed herein. In Figure 12, a sample strain-time-history during FTC is provided and its maximum and minimum strains marked at each cycle as red and blue markers, respectively. Two important values were obtained through these measurements, the first one being the maximum strains of each cycle, which approximately coincide with the initial ambient temperature at which the zero measurement was made, and the average range of strains Δε, which can be used to infer more about the cyclic loading imposed on the anchorage. The maximum and minimum strain readings were observed towards the end of maximum and minimum temperature values, +25 and −15${}^{\circ }$, respectively. The bottom subfigure in Figure 12 shows the maximum strains for both unstressed and prestressed samples at the free-length of the strip. It is evident that although the expected behavior should be towards negative strains (loss of prestressing), the opposite occurred, hinting that the initial assumptions about swelling were correct. An interesting observation drawn from this figure is namely the clustering of strain evolution in two distinct groups. One sample from both unstressed and prestressed blocks converged to a final strain of roughly 100με and 300με, respectively. Although the exact reason for this behavior is not fully understood, it is assumed to be related to the location of each block during FTC exposure. It might be the case that depending on the location, the blocks were exposed to different humidity levels.

Moreover, mean Δε values were calculated for both unstressed and prestressed specimens to obtain the cyclic mechanical load applied on the strip due to the change in temperature. Average strains of 665 and 831 με were calculated for unstressed blocks and 274 and 337 με for the prestressed blocks. The drastic difference between unstressed and prestressed specimens was accredited to the higher bond stiffness achieved by prestressing. These strains can be converted to forces by multiplying with the Young’s modulus provided by the manufacturer (165 GPa) and by the cross-sectional area. This straightforward operation yields an average cyclic load of 6.6 and 9.2 kN for unstressed blocks and 2.7 and 3.3 kN for prestressed blocks, exposed to the bond 120 times.

**Figure 13:**
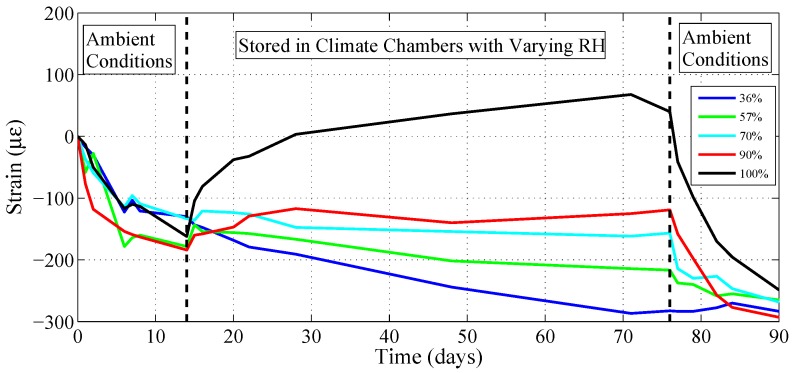
Strain evolution of unstrengthened blocks during: (i) two weeks of storage in the lab environment; (ii) exposure to constant temperature ($20{\;}^{\circ }$C) and a variety of constant RH ranging from 36–100%; (iii) two weeks of storage in the lab environment.

Experiments conducted to measure volumetric effects in concrete proved meaningful based on the strain data during FTC. Average strains measured in three distinct stages are presented in Figure 13. An identical behavior of considerable shrinkage was observed for each block after they were transferred to the laboratory environment from their 90% RH storage. Subsequently, the shrinkage behavior continued for samples that were transferred to 36% and 57% RH chambers. A relative humidity of 70% appeared to have almost no impact, whereas samples stored at 90% and 100% RH experienced swelling. When the blocks were once again transferred to the laboratory, the shrinkage behavior was observed once again, interestingly returning to almost the same value for each case.

### 3.2. Modeling Results

**Figure 14:**
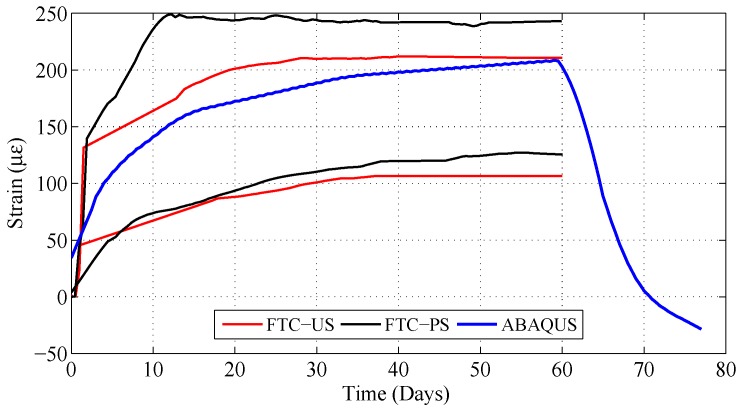
Comparison of FE simulation result with the experimental maximum strain envelope curves at the free-length of the strip. The simulation additionally provides information on the strain evolution after the cycles where the blocks are stored in the lab until lap-shear testing.

Strain values obtained from the block that was exposed to 100% RH were used as the input for the FE model. The first 14 days were not considered since the blocks were strengthened almost before the start of FTC. Hence, the zero measurement was performed at the initiation of humidity exposure. These strains were recreated in the FE-model by providing the measurements as temperature input after a suitable scaling. This scaling was achieved by dividing the measured data into the (arbitrarily) assumed CTE of concrete, 10 μm/m/${}^{\circ }$C in this example. The resulting strain evolution, at the identical location of installed strain sensors, is provided in Figure 14 together with maximum strain envelopes previously presented in Figure 12. Strains from the FE analysis were deemed satisfactory since a significant variance was observed within different samples and swelling of these blocks was not the main purpose of the study.

Measured shrinkage data following humidity exposure were further employed within the simulation to obtain the CFRP strain after FTC exposure until the lap-shear test. It can be observed in Figure 14 that the final strain at the free-length was at a negative value of −28.5 με. This corresponds to a loss in prestressing force of 0.28 kN, concluding that FTC does not induce a significant loss in prestressing force.

**Figure 15:**
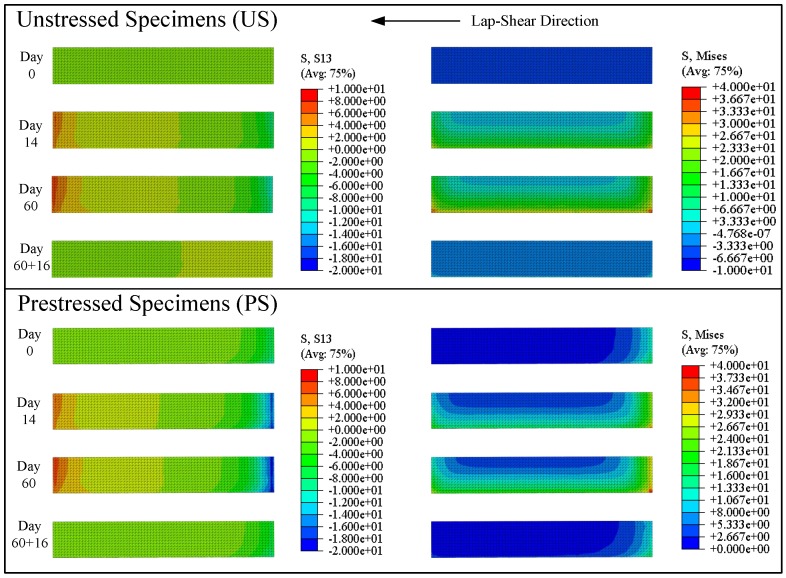
Interfacial shear stresses (**left**) and von Mises stresses (**right**) at the epoxy-concrete interface of unstressed (**top**) and prestressed (**bottom**) blocks at selected time steps. The axis of symmetry is at the top face of each contour plot.

Lastly, a comparison of stresses at the epoxy-concrete interface is made for both unstressed and prestressed samples, computed by the FE simulations and presented in Figure 15. A stress gradient exists before FTC, both in interfacial shear stresses, as well as the von Mises stresses, caused by the prestress-force-releasing step of the gradient anchorage application. Due to the volumetric nature of swelling, an interfacial strain increase (S13) from both ends at the bond is revealed by the FE-model, which indicates a different loading on the bond compared to the typical lap-shear loading. The evolution of the von Mises stresses in both cases shows the same trend.

**Figure A1:**
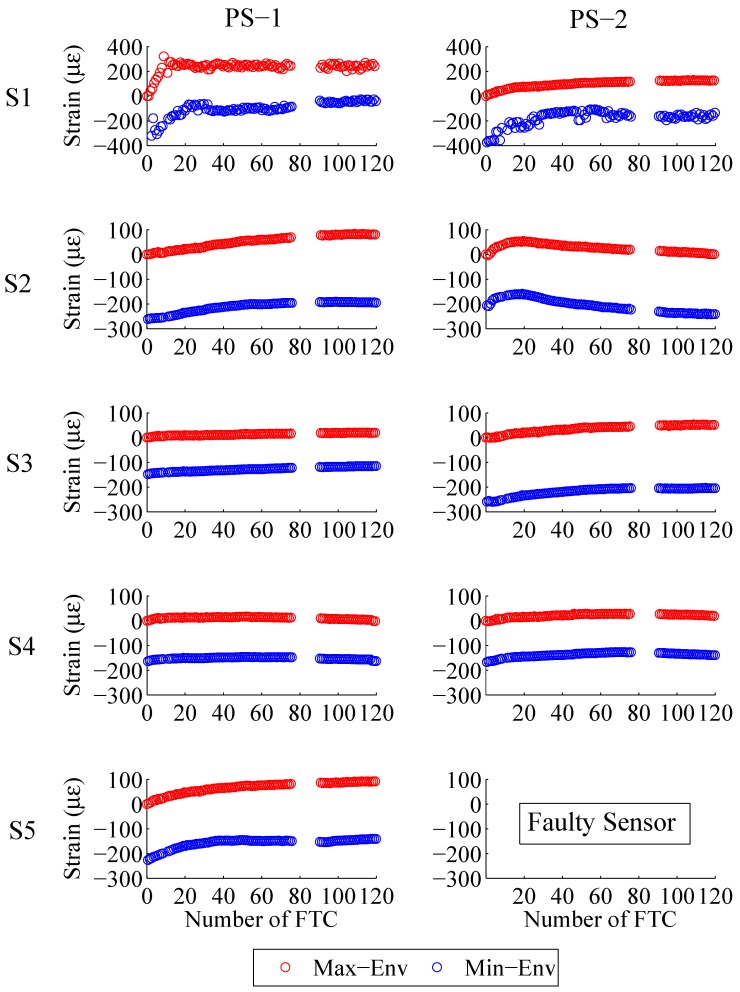
Maximum and minimum strain envelopes of prestressed blocks measured during freeze-thaw cycles. The gap around Cycle 80 is due to the interruption of the data acquisition. Sensor Positions S1–S5 are provided in Figure 4.

A similar observation was also made experimentally from the FOS sensors attached on the CFRP strip along the bonded area (Denoted as S1–S5 in Figure 4), as shown in Figure A1. Analogous to the interfacial stresses, the maximum strain envelope shows higher values for sensors closer to the extremes of the bond (S2 and S5) compared to the inner locations (S3 and S4). Similarly, minimum strain envelopes are also affected to a greater extent at locations closer to the extremes of the bond.

## 4. Conclusions

An experimental and numerical investigation on the behavior of a non-mechanical unstressed/prestressed CFRP anchorage during and after FTC was conducted. The obtained results indicated a deteriorative effect of FTC on the bond, and the main findings of the study are summarized below.

- Freeze-thaw cycles in combination with humidity provoked a reduction in the residual anchorage resistance, maximum slip capacity, as well as elastic bond stiffness.
- Degradation became more significant in the case of prestressed blocks, especially for the residual load carrying capacity, indicating that synergistic exposure to mechanical and environmental loading led to a more severe deterioration.
- A shift in failure mode from concrete substrate to an epoxy-concrete interface failure was observed both in unstressed and prestressed blocks that were subjected to FTC.
- A trend towards tensioning was observed at the free-length of the strips during FTC. This was later explained via the swelling of concrete due to exposure to humidity. This effect was reversed after the blocks were transferred to the laboratory, in which the strains returned back to initial state via shrinkage.
- The average difference between minimum and maximum strain values during FTC revealed that the strip exerted a considerably high cyclic loading on the bond, with values ranging 6.6–9.2 kN for unstressed and 2.7–3.3 kN for the prestressed blocks.
- An FE model was constructed to observe the effect of swelling during FTC on the bond and further derive the prestressing force after FTC. An agreeable match was observed with the use of a temperature field as the input to emulate the volumetric swelling behavior. An insignificant amount of loss was computed for the prestressing force after FTC. The effect of asymmetric stress states, both due to prestressing, as well as swelling was visualized.

In light of these findings, future research focus could be steered towards two distinct directions. The first path would be methodologies that embrace the deterioration and involve the development of techniques that incorporate such effects during the design process. It needs to be kept in mind that the results presented herein were from a single type of a commercially available epoxy; hence, a similar experimental campaign would be needed to obtain the deterioration effect quantitatively of another epoxy resin. A second path would be to investigate ways to enhance the bonding properties of epoxy against humidity exposure. In recent studies, silane coupling agents have been successfully utilized to decrease the effect of bond degradation after hygrothermal aging [30,31]. Incorporation of such additives in the epoxy mixture could potentially inhibit the interface from becoming the weakest link of EBR systems following humidity exposure.

## Figures and Tables

**Table 1:** Results obtained from lap-shear tests, both for unstressed and prestressed specimens.

	Unstressed		Prestressed	
	REF-US	FTC-US	REF-PS	FTC-PS
$F_{u}$ (kN)	28.23	20.53	33.14	21.57
$\Delta F_{u}$ (kN)	28.23	20.53	25.14	13.57
$s_{f,B}$ (mm)	0.23	0.13	0.22	0.093
$\frac{\Delta F_{u,ftc}}{\Delta F_{u,ref}}$ (-)	0.73		0.54	
$\frac{F_{el}}{s_{f,B,el}}$ (kN/mm)	316.0	289.8	1057.4	716.8

## Abbreviations

The following abbreviations are used in this manuscript:

(P)EBR	(Prestressed) externally-bonded reinforcement
RC	Reinforced concrete
CFRP	Carbon fiber-reinforced polymer
FTC	Freeze-thaw cycles
DIC	Digital image correlation
US	Unstressed
PS	Prestressed
FOS	Fiber optic (strain) sensors
RH	Relative humidity
FE	Finite element
CTE	Coefficient of thermal expansion
BC	Boundary condition
